# Preparing high-quality chromosome spreads from *Crocus* species for karyotyping and FISH

**DOI:** 10.1186/s13039-025-00706-7

**Published:** 2025-02-20

**Authors:** Abdullah El-nagish, Susan Liedtke, Sarah Breitenbach, Tony Heitkam

**Affiliations:** 1 Department of Biology, Institute of Biology I, RWTH Aachen University, Worringer Weg 3, 52074 Aachen, Germany; 2 Botany and Microbiology Department, Faculty of Science, Sohag University, Sohag, 82524 Egypt; 3 Faculty of Biology, Institute of Botany, Technische Universität Dresden, 01069 Dresden, Germany

**Keywords:** Hydroxyurea-colchicine method, Nitrous oxide, Hydroxyquinoline, Ice Water pretreatment, Metaphase index, Saffron, *Crocus sativus*, Fluorescent in situhybridization

## Abstract

**Background:**

The saffron-producing *Crocus sativus* (L.) and its wild relative *C. cartwrightianus* (Herb.) are key species for understanding genetic evolution in this genus. Molecular-cytogenetic methods, especially fluorescent in situ hybridization (FISH), are essential for exploring the genetic relationships in this genus. Yet, preparing high-quality chromosomes for FISH analysis across *Crocus* species remains difficult. A standardized protocol for achieving clear and well-separated mitotic chromosomes is still lacking. This study aimed to assess the effectiveness of pretreatments with four chromosome synchronization methods for optimal chromosome spread preparation in *Crocus*. Root tips of different *Crocus* species were treated with four chromosome preparation methods namely hydroxyurea-colchicine (HC), nitrous oxide (NO), hydroxyquinoline (HQ), and ice water (IW) pretreatments to investigate their effectiveness in producing high-quality mitotic chromosome spreads. Metaphases obtained by the four methods were analyzed to assess their quality and metaphase index.

**Results:**

Evaluation of 22,507 cells allowed us to confidently recommend a protocol for *Crocus* chromosome preparation. Among the methods, ice water pretreatment yielded the highest metaphase index (2.05%), more than doubling the results of HC (1.08%), NO (1.15%), and HQ (1.16%). Ice water-treated chromosomes exhibited better chromosome morphology, with relatively proper size, and non-overlapping chromosomes that were optimal for FISH analysis. Ice water pretreatment was also applied to *C. cartwrightianus*, the diploid progenitor of *C.* *sativus*, where it demonstrated similar efficacy. DAPI staining of chromosomes in both species allowed for clear visualization of intercalary and terminal heterochromatin. FISH analysis using 18S-5.8S-25S and 5S rDNA probes confirmed the utility of IW-prepared chromosome spreads for cytogenetic studies.

**Conclusions:**

We strongly recommend ice water pretreatment as a suitable and effective method for obtaining many metaphase spreads of high-quality in *C. sativus* and related species, particularly for applications involving a detailed cytogenetic analysis.

## Background

The genus *Crocus* (Iridaceae) comprises around 250 species widely distributed over a wide range of climatic areas [1, 2] and is known for its variable chromosome numbers [3–5]. Among the species of *Crocus*, only *C.* *sativus* is used as a crop and thus receives the most attention. *C.* *sativus* is the source of saffron, one of the highest priced spices of the world, processed from dried stigmas of manually harvested flowers. It is a cash crop for agriculture-based communities living off marginal areas in Iran, North Africa, countries surrounding the Mediterranean basin, and Kashmir. Despite its economic relevance, we are just beginning to understand the genomic and chromosomal constitution of saffron crocus and related species.

*C. sativus* is a male-sterile triploid species harboring eight chromosome triplets (2n = 3x = 24) and having a genome size of 1C = 3.45 Gbp [6]. Due to its triploidy, saffron crocuses can only be propagated vegetatively. As all saffron accessions around the globe have a similar genome, it is generally accepted that triploid saffron emerged only once [3, 7–11]. We and other groups recently showed that cytotypes of *Crocus cartwrightianus* have been the sole precursors of saffron’s triploidy, and that its emergence can be traced to the Aegean Bronze Age in Greece [3, 4, 12, 13]. *C.* *cartwrightianus* is a diploid species (2n = 16) with high genetic diversity [3, 4, 12]. Nevertheless, despite these recent insights into the origin of saffron crocus, the *C.* *cartwrightianus* cytotypes that may enable targeted re-breeding and improvement of saffron traits have not yet been identified. Similarly, the origin of the individual chromosomes within saffron’s chromosome triplets is still unclear, especially as one chromosome is heteromorphic [14]. However, genomic and cytogenetic analyses in the genus *Crocus* may provide detailed insights into the chromosome structure of *Crocus* species, but robust and widely applicable protocols are still lacking.

Fluorescent in situ hybridization (FISH) is a powerful cytogenetic technique to study structure and function of chromosomes, polyploidy and genome evolution. In particular, the physical mapping of tandemly repeated DNA sequences provides informative cytogenetic landmarks for unequivocal chromosome identification in many plant species [15–19]. The first in situ hybridizations along *Crocus* chromosomes already showed the potential of repeat probes in this genus, yielding a range of distinct signals and allowing first chromosome assignments [20–22]. Recently, we developed a karyotyping mix for *Crocus* species that is composed of six tandem repeat probes [4] and that opens the *Crocus* genus for comprehensive molecular-cytogenetic analyses to clarify the genetic details of saffron crocus’ ancestry. However, streamlining FISH analysis across a range of species, cytoypes and accessions requires a robust protocol for properly dispersed mitotic chromosomes for its application. Despite being studied cytogenetically for several decades, species of the *Crocus* genus still remain challenging targets for chromosome preparation. Usually, due to the strict annual growth and the small size of the plant, material is limited, especially of wild accessions. In addition, as the chromosomes are large, some preparation techniques such as dropping [23–26] are not recommended. Therefore, a comparative study testing different methods to obtain high-quality mitotic chromosomes from *Crocus* species is needed.

Here, using the crop plant *C.* *sativus* for its high economic value and *C.* *cartwrightianus* for its scientific interest, we compared the effectiveness of four chromosome fixation methods for obtaining *Crocus* chromosome spreads. We use (1) the hydroxyurea-colchicine method, (2) the nitrous oxide method, (3) the hydroxyquinoline method, and (4) ice water treatments and analyze them for their yield in obtaining properly spread mitotic chromosomes and mitotic index.

## Methods

### Plant materials and time of harvest

We used root tips of *C.* *sativus* (corms collected commercially), *C.* *cartwrightianus HKEP 1517* and *C.* *cartwrightianus* (Attica S FB19-63 (2)) was provided by F. Blattner and D. Harpke (IPK Gatersleben, Gatersleben, Germany). All plants were grown under glasshouse conditions. Root tips were collected in the early morning hours (07.00–08.00).

### Experimental design

Root tips of *C.* *sativus* were subjected to four different pretreatments techniques. Each was repeated six times:

1. *Hydroxyurea-colchicine method (HC)* Corms with roots were incubated for 18 h in liquid, 0.5 × LM medium containing 1.25 mM hydroxyurea. The material was kept in 125 mL Erlenmeyer flasks placed on an orbital shaker at 150 rpm at room temperature. After three rinses with 0.5 × LM medium without hydroxyurea, the material was incubated for 6 h in fresh medium followed by treatment with the medium containing 0.6% (w/v) colchicine for 20 h [27]. Treated root tips were excised and fixed in a 3:1 (v/v) ethanol: acetic acid solution for 24 h at 4 °C.
2. *Nitrous Oxide (NO)* Root tips were incubated in a pressure-tolerant cylinder, with nitrous oxide gas applied for 45 min at room temperature at 10 bar [28]. After this, the root tips were fixed in a 3:1 (v/v) methanol: acetic acid solution for 24 h at 4 °C [29].
3. *Hydroxyquinoline (HQ)* Roots from individual corms were collected, pretreated with 2 mM hydroxyquinoline for 5 h and fixed in a 3:1 methanol: acetic acid [4].
4. *Ice water (IW)* Corms with roots were placed in container filled with ice water, kept inside a refrigerator at 4∘ C for 18 h. Roots of 2–3 cm long were cut from and were fixed in methanol: acetic acid (3: 1) for 2 h at 4 °C, fresh fixative was add and kept for 24 h at 4 °C.

### Protocol of chromosome preparation from *Crocus* material

#### Enzyme treatment of root tips

1. Wash root tips 1 × in dist. H_2_O for 5–10 min, 2 × in 4 mM citrate buffer (4 mM citric acid and 6 mM sodium citrate), pH 4.5 for 5 min each.
2. Dissect meristematic tips using a sharp scalpel and transfer them into a petri dish with 20–30 μl of enzyme mixture. The enzyme solution consists of 2% (w/v) cellulase from *Aspergillus niger*, 4% (w/v) cellulase ‘Onozuka R10′ from *A. niger*, 2% (w/v) hemicellulase from *A. niger*, 0.5% (w/v) pectolyase from *Aspergillus japonicus* and 20% (v/v) pectinase from *A. niger* in citrate buffer.
3. Incubate at 37 °C for 2.0–2.5 h depending on species (Table 1).

**Table 1:** Incubation times of different *Crocus* root tips for all chromosome preparation protocols

Species/Accession	Incubation time in enzyme mixture	Incubation time on hot plate
*C. sativus*	2.30 h	60 s
*C. cartwrightianus* HKEP 1517	2.00 h	30 s
*C. cartwrightianus* (Attica S FB19-63 (2))	2.15 h	30 s

#### Spread preparation

1. Single root tips were transferred onto slides, macerated in 50–60 μl of 45% acetic acid with needles for 120 sec. “Methods”. Add an extra drop of 45% acetic acid, mix with a needle, spread on a hot surface at 55 °C for 30–60 s depending on species (Table 1).

#### Spread fixation

1. Surround with drops of freshly prepared fixative (3:1 methanol:acetic acid), dropwise fresh fixative on top of the slide as well.
2. Let the fixative run down, rinse with more fixative.
3. Air-dry the slides.
4. Store slides in a Coplin jar in a freezer until usage.

### Assessing the quality of the mitotic chromosome spreads via phase contrast and fluorescent microscopy

The chromosome preparations were assessed via phase contrast and fluorescent microscopy. For the latter, mitotic chromosome spreads were prepared according to the protocol above. Slides were equilibrated in 4 × SSC/0.2% Tween 20 for 5 min at 37 °C. Excess liquid was carefully removed, then, 20 μl DAPI-Citifluor AF1 were added on each slide and covered with glass cover.

Wide-field imaging was performed with a Zeiss Axioimager M1 UV epifluorescence microscope with appropriate filters, and equipped with an ASI BV300-20A camera coupled with the Applied Spectral Imaging software (Applied Spectral Imaging, Carlsbad, CA, USA). The images were processed with Adobe Photoshop CS5 software (Adobe Systems, San Jose, CA, USA) using only contrast optimization, Gaussian and channel overlay functions affecting the whole image equally.

The quality of the mitotic chromosome spreads was evaluated based on chromosome morphology, the absence of overlapping chromosomes, and the clarity of the spread from any debris on the slide. Chromosome spreads were considered high quality if they exhibited well-delineated, intact chromosomes with minimal background noise, which allowed for easy and accurate identification of individual chromosomes eventually facilitating accurate FISH signal detection. Metaphase index was calculated as the percentage of cells at metaphase stage. Based on the quality of the chromosome preparations and the highest mitotic indices, the best protocol was selected and tested for suitability for wild *Crocus* accessions, using two *C.* *cartwrightianus* accessions as use cases.

### Probe labeling and fluorescent in situ hybridization

The probe “18SrRNAgene_Bv_probe1” [30] was used for the detection of the rDNA and was labeled with biotin-11-dUTP (Dyomics) by PCR and detected by Streptavidin-Cy3 (Sigma-Aldrich). The probe pXV1 [31] for the 5S rRNA gene was labeled with digoxygenin-11-dUTP by PCR and detected by anti-digoxigenin–fluorescein isothiocyanate (FITC; both from Roche Diagnostics). The hybridization procedure was performed as described previously [31]. Chromosomes were counterstained with DAPI (Honeywell, Charlotte, NC, USA). Prior to FISH, according to the amount of cytoplasm visible under light microscope, we pre-treated the slides with 100 μg/ ml RNase in 2 × SSC for 30 min, followed by 200 μl of 10 μg/ ml pepsin in 10 mM HCl for 15 to 30 min.

## Results and discussion

### Ice water pretreatment is most effective for obtaining metaphase spreads of high-quality in *Crocus*

To obtain chromosome spreads ideal for karyotyping, FISH and various other cytogenetic analysis, the use of appropriate arresting agent is required. These agents influence chromosome morphology, condensation, and the quality of chromosome spreads. The mechanisms by which these agents work include altering cytoplasmic viscosity, disrupting spindle fiber formation, and interfering with chromosome condensation [32, 33]. These effects ultimately impact the number of dividing cells and their chromosomal morphology and arrangement.

**Fig. 1:**
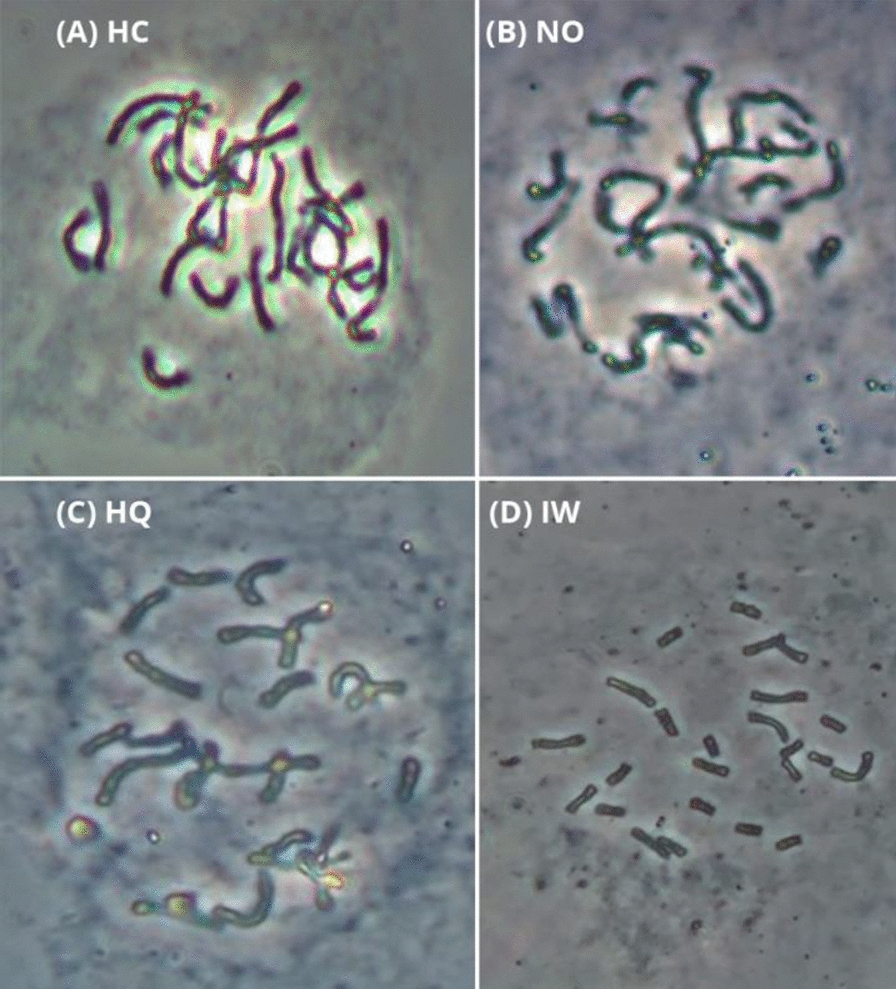
Mitotic chromosome of *C. sativus*, obtained with each of the four treatments: HC (**A**), NO (**B**), HQ (**C**), and IW (**D**). The spreads shown in this figure were taken by phase contrast microscope (Zeiss Axioskop 40) at a magnification power of 400x.

In this study, we evaluated four different pretreatments (Hydroxyurea-colchicine (HC), Nitrous Oxide (NO), Hydroxyquinoline (HQ), and Ice Water (IW)) were compared for their effectiveness in obtaining properly dispersed mitotic chromosomes of *C. sativus*, focusing on improving the quality of spreads and their suitability for subsequent FISH analysis. These methods were applied to root tips of *C.* *sativus* and *C.* *cartwrightianus* species. In total, 22,507 cells were evaluated and representative metaphases were selected for illustration (Fig. 1, Table 2).

**Table 2:** Comparison of metaphase indices in four methods used for chromosome preparation from *C.* *sativus* materials

Method	No. of cells analyzed	No. of metaphases	Metaphase index (%)
Hydroxyurea-colchicine method (HC)	3717	40	1.08
Nitrous oxide (NO)	4714	54	1.15
Hydroxyquinoline (HQ)	6400	74	1.16
Ice water (IW)	7676	157	2.05

Comparing all four methods, HC (Fig. 1A) yielded the lowest metaphase index (1.08%). HC-derived metaphases usually featured chromosomes that were difficult to count due to overlapping of chromosome arms. Hence, using HC for chromosome preparation and FISH analysis of *Crocus* is not recommended (Fig. 1A, Table 2). HU induces cell synchronization at the stage of DNA synthesis. Hydroxyurea is an inhibitor, acting on the ribonucleotide reductase leading to inhibition of DNA synthesis, arresting cells in the S-phase. In contrast, colchicine disrupts microtubules, preventing mitotic spindle formation and arresting cells in metaphase. Although the hydroxyurea/colchicine combination often used for cell synchronization [27, 34, 35], our results suggest that it is not suitable for *Crocus*, likely due to its inefficiency in chromosome condensation or factors related to concentration, exposure time, and species-specific features. Further optimization of these parameters is needed to improve HU effectiveness in *Crocus*.

Similar to HC, the chromosome spreads obtained by Nitrous Oxide (NO) were difficult to distinguish also due to overlapping chromosome arms resulting in limited effectiveness in producing high-quality chromosome spreads (Fig. 1B, Table 2), complicating accurate identification and analysis. NO likely interferes with mitotic spindle formation, but the lack of effective chromosome condensation reduced the resolution of the spreads. The exact cause of the low-quality chromosomal spreads with NO in *Crocus* is unclear. Nitrous oxide might be toxic in *Crocus*, causing cell cycle arrest before metaphase, as indicated by the low metaphase index. Unlike other pretreatments, NO is not known to affect spindle fiber formation; rather, it prevents proper chromosome alignment along the metaphase plate, hindering spindle fiber attachment [36].

The HQ method (Fig. 1C, Table 2) yielded comparable results, with a metaphase index of 1.16%, slightly higher than NO but still insufficient for obtaining consistently clear, non-overlapping chromosomes. HQ, known to alter cytoplasmic viscosity [32, 33, 37], likely interferes with chromosome condensation, but may not be as efficient as other agents, resulting in a lower metaphase index and suboptimal spread quality.

Weighing all four methods against each other, IW yielded the highest metaphase index (2.05%). We conclude that this pretreatment arrested the cells in metaphase twice more often than the other three methods (Table 2). Moreover, this method is preferred in terms of chromosome morphology, if the chromosomes are to be counted or analyzed by FISH procedure, as they were of preferred shape and length with no overlapping (Fig. 1D).

The increased the metaphase index and preventing overlapping of chromosome arms is evidence that the chilling stress stops DNA synthesis and mitotic activity and inhibits formation of microtubules [38, 39], leading to a better separation of single chromosomes suitable for further analyses.

### Ice water pretreatment is also effective for wild crocus species and allows following the chromosomes through cell cycle

**Fig. 2:**
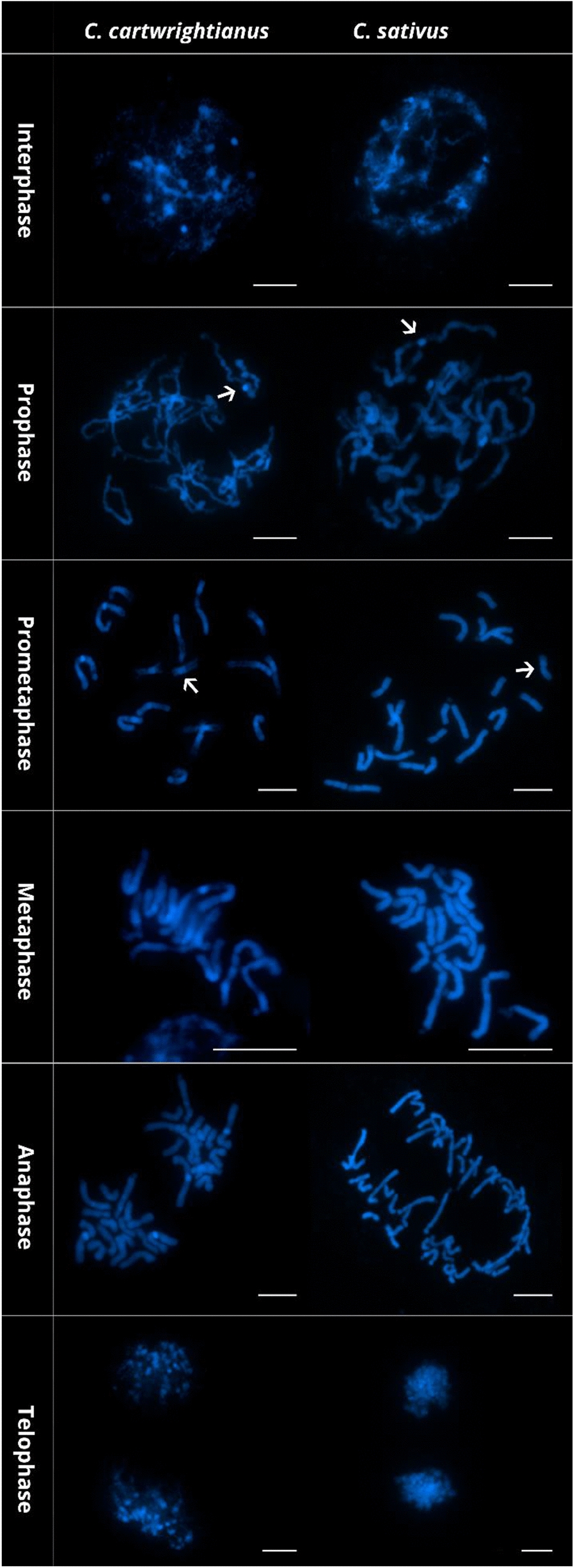
DAPI-stained chromosome spreads of *C.* *cartwrightianus* and *C. sativus*. The scale bar equals 10 µm. Arrows indicate the centromeric constriction

To test the most effective method also for wild *Crocus* species, we applied the ice water pretreatment also to *C. cartwrightianus*, the diploid progenitor species of *C.* *sativus*. Using IW pretreatment, chromosomes from both species, *C.* *sativus* and *C. cartwrightianus*, were easily stained with DAPI, resulting in clear, well-resolved chromosome spreads that allowed for detailed visualization of each stage of mitosis (Fig. 2). The preparations showed high-quality chromosome morphology, with distinct and non-overlapping chromosomes across the mitotic phases, facilitating accurate structural analysis as shown later by the FISH analysis. In the DAPI-stained images of both *C. sativus* and *C.* *cartwrightianus* prepared using the IW method, all stages of mitosis (interphase, prophase, prometaphase, metaphase, anaphase, and telophase) were clearly observed. Each stage displayed its characteristic features of chromosome condensation and organization (Fig. 2).

The DAPI-staining of both *C.* *sativus* and *C.* *cartwrightianus* chromosomes enabled the identification of intercalary and terminal heterochromatin and of the mostly weakly stained centromeres, often also visible as a constriction (Fig. 2; arrowed).

### Metaphases resulting from ice water pretreatment are well-suited for FISH follow-up

**Fig. 3:**
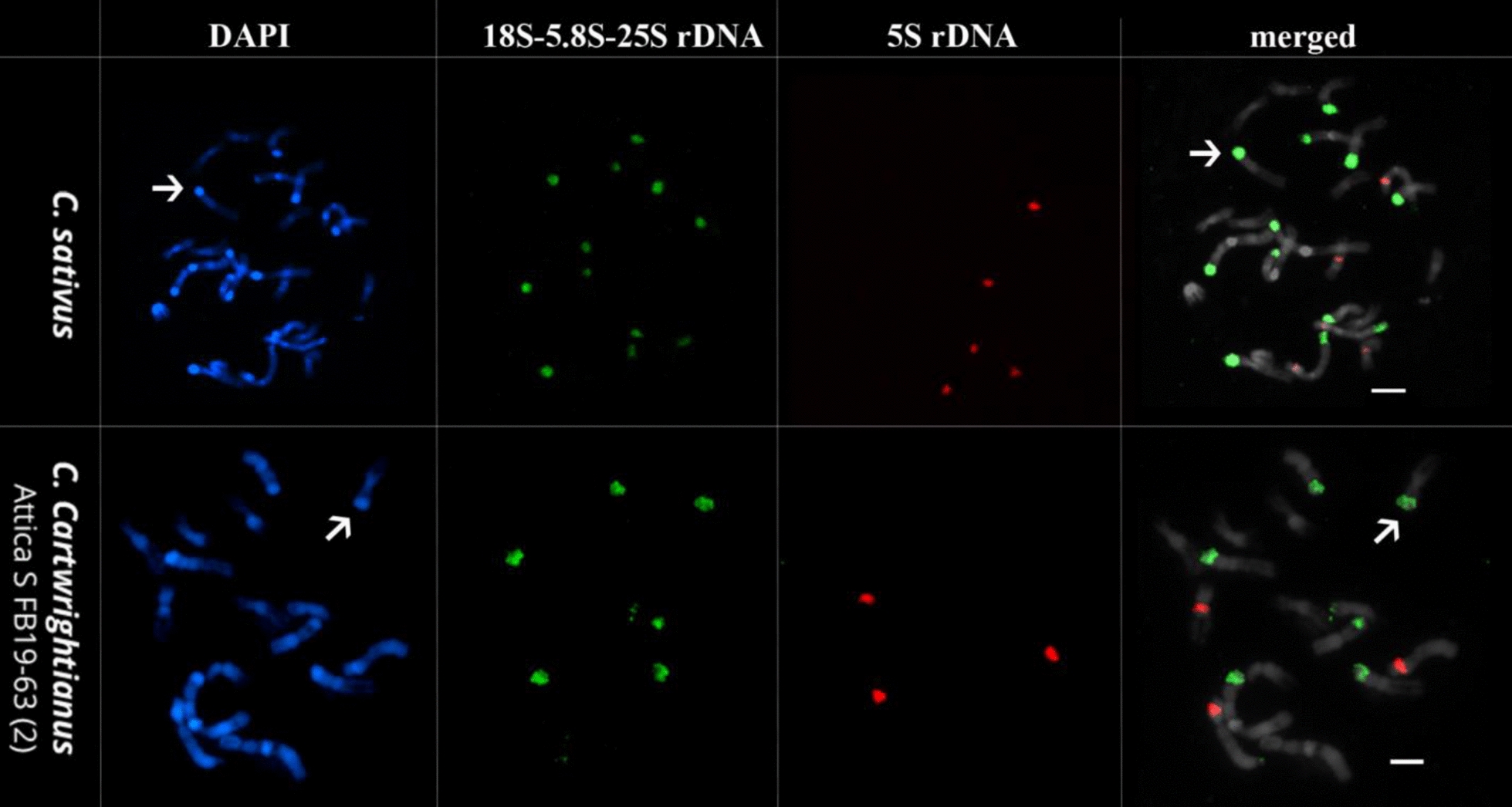
Fluorescent in situ hybridization (FISH) of *C.* *sativus* and *C.* *cartwrightianus* (name given in each panel). DAPI-stained mitotic chromosomes are shown in blue. Probes used are 18S-5.8S-25S rRNA genes (green), and 5S rRNA genes (red). Arrows indicate exemplary DAPI-positive chromosome regions, which are shown to be enriched in rDNA.

Chromosome spreads obtained from root tips prepared by the IW method were evaluated for their applicability for FISH analysis using 18S-5.8S-25S and 5S rDNA probes (Fig. 3).

As presented (Fig. 3), the chromosome spreads produced with IW were highly useful for follow-up analysis by FISH. In this example, the genetic identity can be assigned to the large terminal chromosome regions, which are strongly condensed and detectable as DAPI-positive blocks. These consist mostly of rRNA genes (Fig. 3, arrowed, Table 3). In *C. sativus*, we detected 12 arrays of 18S-5.8S-25S rRNA genes, including five major sites and seven moderate sites (Fig. 3, arrowed), and five major 5S rRNA gene arrays (Fig. 3, arrowed). Eight hybridization signals were detected in metaphase chromosomes of *C.* *cartwrightianus* using 18S-5.8S-25S rDNA probe, of which six were strong signals while the remaining two were weak signals. FISH analysis using 5S rDNA revealed five moderate signals in *C.* *sativus* and three strong signals in *C. cartwrightianus*. The number, relative signal strength and presence on chromosomes of the two rDNA sites are listed in Table 3.

**Table 3:** Hybridization of cytogenetic landmarks in *C.* *sativus* and *C.* *cartwrightianus* using two rDNA probes

Tandem repeat	Species	Number of sites				Number of labeled chromosomes
		major	moderate	minor	total	
18S-5.8S-25S rRNA genes	*C. sativus*	5	7	0	12	12
	*C. cartwrightianus*	6	0	2	8	8
5S rRNA genes	*C. sativus*	0	5	0	5	5
	*C. cartwrightianus*	3	0	0	3	3
Total number		14	12	2	28	

## Conclusion

Summarizing, we present a comprehensive comparison of four methods (HC, NO, HQ, and IW) for preparing mitotic chromosome spreads in *Crocus sativus*. The results indicate that ice water pretreatment (IW) is more suitable, yielding the highest metaphase index (2.05%) and providing the best chromosome morphology for further analysis. In contrast, HC, NO, and HQ yielded lower metaphase indices and produced chromosomes with poor morphology, making them less suitable for cytogenetic studies in *Crocus*. The unsuitability of the three techniques may result from non-optimized parameters for *Crocus*. Future studies to optimize the parameters of these techniques for *Crocus* are needed. IW pretreatment was also equally well with *C. cartwrightianus*, the diploid wild progenitor species.

The suitability of this method for cytogenetic analysis across different *Crocus* species was demonstrated by a) enabling us of following the different stages of mitosis in both species with a clear identification of DAPI-stained metaphase chromosomes that provided detailed insights into chromosomal structure, b) the successful FISH hybridization to chromosomes enabling the visualization of key chromosomal features such as heterochromatin and centromeres in both cultivated and wild crocus species.

## Data Availability

Not applicable.

## Abbreviations

DAPI: 4,6-Diamidino-2-phenylindole
FISH: Fluorescence in situ hybridization
Hq: 8-Hydroxyquinoline
rDNA: Ribosomal DNA
RT: Room temperature
DW: Distilled water
SSC: Saline sodium citrate

## References

[1] Rukšāns J. The world of crocuses. Latvian Academy of Sciences; 2017.

[2] Pastor-Férriz T, De-los-Mozos-Pascual M, Renau-Morata B, et al. Ongoing evolution in the genus *Crocus*: diversity of flowering strategies on the way to hysteranthy. Plants. 2021;10:477. 10.3390/plants10030477. 33802494 10.3390/plants10030477 PMC7999489

[3] Nemati Z, Harpke D, Gemicioglu A, et al. *Crocus sativus* is an autotriploid that evolved in Attica (Greece) from wild *Crocus cartwrightianus*. Mol Phylogenet Evol. 2019;136:14–20. 30946897 10.1016/j.ympev.2019.03.022

[4] Schmidt T, Heitkam T, Liedtke S, et al. Multi-color chromosome identification unravels the autotriploid nature of saffron (*Crocus sativus*) as a hybrid of wild *Crocus cartwrightianus* cytotypes. New Phytol. 2019;222(4):1965–80. 30690735 10.1111/nph.15715

[5] Raca I, Blattner FR, Waminal NE, et al. Disentangling *Crocus* series *Verni* and its polyploids. Biology. 2023;12(2):303. 36829579 10.3390/biology12020303 PMC9953621

[6] Brandizzi F, Grilli CM. Flow cytometric analysis of nuclear DNA in *Crocus sativus* and allies (Iridaceae). Plant Syst Evol. 1998;211(3):149–54.

[7] Rubio-Moraga A, Castillo-López R, Gómez-Gómez L, et al. Saffron is a monomorphic species as revealed by RAPD, ISSR, and microsatellite analyses. BMC Res Notes. 2009;2(1):1–5. 19772674 10.1186/1756-0500-2-189 PMC2758891

[8] Fluch S, Hohl K, Stierschneider M, et al. *Crocus sativus* L.—Molecular evidence on its clonal origin. Acta Hortic. 2010;850:41–6.

[9] Siracusa L, Gresta F, Avola G, et al. Agronomic, chemical, and genetic variability of saffron (*Crocus sativus* L.) of different origin by LC-UV–vis-DAD and AFLP analyses. Genet Resour Crop Evol. 2013;60(2):711–21.

[10] Babaei S, Talebi M, Bahar M, et al. Analysis of genetic diversity among saffron (*Crocus sativus*) accessions from different regions of Iran as revealed by SRAP markers. Sci Hortic. 2014;171:27–31.

[11] Alsayied NF, Fernández JA, Schwarzacher T, et al. Diversity and relationships of *Crocus sativus* and its relatives analysed by inter-retroelement amplified polymorphism (IRAP). Ann Bot. 2015;116(3):359–68. 26138822 10.1093/aob/mcv103 PMC4549961

[12] Nemati Z, Blattner FR, Kerndorff H, et al. Phylogeny of the saffron-*Crocus* species group, *Crocus* series *Crocus* (Iridaceae). Mol Phylogenet Evol. 2018;127:891–7. 29936028 10.1016/j.ympev.2018.06.036

[13] Kazemi-Shahandashti SS, Mann L, El-Nagish A, et al. Ancient artworks and *Crocus* genetics both support saffron’s origin in early Greece. Front Plant Sci. 2022;13:374. 10.3389/fpls.2022.834416 PMC8913524 35283878

[14] Agayev YM, Zarifi E, Fernández JA. A study of karyotypes in the *Crocus sativus* L. aggregate and origin of cultivated saffron. Acta Hortic. 2010;850:47–54.

[15] Gindullis F, Desel C, Galasso I, et al. The large-scale organization of the centromeric region in *Beta* species. Genome Res. 2001;11(2):253–65. 11157788 10.1101/gr.162301 PMC311043

[16] Findley SD, Cannon S, Varala K, et al. A fluorescence *in situ* hybridization system for karyotyping soybean. Genetics. 2010;185(3):727–44. 20421607 10.1534/genetics.109.113753 PMC2907198

[17] Begum R, Zakrzewski F, Menzel G, et al. Comparative molecular cytogenetic analyses of a major tandemly repeated DNA family and retrotransposon sequences in cultivated jute *Corchorus* species (Malvaceae). Ann Bot. 2013;112(1):123–34. 23666888 10.1093/aob/mct103 PMC3690992

[18] Heitkam T, Petrasch S, Zakrzewski F, et al. Next-generation sequencing reveals differentially amplified tandem repeats as a major genome component of Northern Europe’s oldest *Camellia japonica*. Chromosome Res. 2015;23:791–806. 26582634 10.1007/s10577-015-9500-x

[19] Křivánková A, Kopecký D, Stočes Š, Doležel J, Hřibová E. Repetitive DNA: a versatile tool for karyotyping in *Festuca pratensis* Huds. Cytogenet Genome Res. 2017;151(2):96–105. 28334706 10.1159/000462915

[20] Ørgaard M, Jacobsen N, Heslop-Harrison JS. The hybrid origin of two cultivars of *Crocus* (Iridaceae) analysed by molecular cytogenetics including genomic Southern and *in situ* hybridization. Ann Bot. 1995;76(3):253–62.

[21] Frello S, Heslop-Harrison JS. Chromosomal variation in *Crocus vernus* Hill (Iridaceae) investigated by *in situ* hybridization of rDNA and a tandemly repeated sequence. Ann Bot. 2000;86(2):317–22.

[22] Frello S, Ørgaard M, Jacobsen N, et al. The genomic organization and evolutionary distribution of a tandemly repeated DNA sequence family in the genus *Crocus* (Iridaceae). Hereditas. 2004;141(1):81–8. 15383076 10.1111/j.1601-5223.2004.01839.x

[23] Kato A, Lamb JC, Albert PS, Danilova T, Han F, Gao Z, Findley S, Birchler JA. Chromosome painting for plant biotechnology. Methods Mol Biol. 2011;701:67–96. 21181525 10.1007/978-1-61737-957-4_4

[24] Aliyeva-Schnorr L, Ma L, Houben A. A fast air-dry dropping chromosome preparation method suitable for FISH in plants. J Vis Exp. 2015;106:53470. 10.3791/53470 PMC4694022 26709593

[25] Schmidt T, Weber B, Klekar J, et al. Preparation of mitotic chromosomes using the dropping technique. In: Methods Mol Biol: Plant Cytogenetics and Cytogenomics. Heitkam T, Garcia S, editors. Springer Nature, US; 2023. 10.1007/978-1-0716-3226-0_8 37335474

[26] Heitkam T, Garcia S, editors. Plant cytogenetics and cytogenomics: methods & protocols. Methods Mol Biol. Vol 2672. Humana, New York; 2023.

[27] Nkongolo KK, Klimaszewska K. Karyotype analysis and optimization of mitotic index in *Picea mariana* (black spruce) preparations from seedling root tips and embryogenic cultures. Heredity. 1994;73(1):11–7.

[28] Kato A. Air drying method using nitrous oxide for chromosome counting in maize. Biotech Histochem. 1999;74:160–6. 10416789 10.3109/10520299909047968

[29] Vrána J, Cápal P, Šimková H, Karafiátová M, Čížková J, Doležel J. Flow analysis and sorting of plant chromosomes. Curr Protoc Cytom. 2016;78(1):5–3. 10.1002/cpcy.9 27723090

[30] Sielemann K, Schmidt N, Guzik J, et al. Pangenome of cultivated beet and crop wild relatives reveals parental relationships of a tetraploid wild beet. bioRxiv. 2023. 10.1101/2023.06.28.546919.

[31] Schmidt T, Schwarzacher T, Heslop-Harrison JS. Physical mapping of rRNA genes by fluorescent *in-situ* hybridization and structural analysis of 5S rRNA genes and intergenic spacer sequences in sugar beet (Beta vulgaris). Theor Appl Genet. 1994;88(6):629–36. 24186156 10.1007/BF01253964

[32] Sharma A. Plant chromosomes. Boca Raton: CRC Press; 2019.

[33] Singh RJ. Plant cytogenetics. Boca Raton: CRC Press; 2016.

[34] Pan WH, Houben A, Schlegel R. Highly effective cell synchronization in plant roots by hydroxyurea and amiprophos-methyl or colchicine. Genome. 1993;36(2):387–90. 18469996 10.1139/g93-053

[35] Shen J, Xu J, Chen J, Zheng R, Shi J. Cell synchronization and isolation of chromosomes from Chinese fir root tips for flow cytometric analysis. Biotech letters. 2015;37:1309–14. 10.1007/s10529-015-1800-x 25953501

[36] Brinkley BR, Rao PN. Nitrous oxide: effects on the mitotic apparatus and chromosome movement in HeLa cells. J Cell Bio. 1973;58(1):96–106. 4726309 10.1083/jcb.58.1.96 PMC2109015

[37] Lima MG, Silveira GL, Techio VH, Andrade-Vieira LF. Effects of three antimitotic agents on karyotype of *Allium cepa* L. and *Lactuca sativa* L.: two plant model species for cytogenotoxic assessments. S Afr J Bot. 2019;125:244–50.

[38] Carter JV, Wick SM. Irreversible microtubule depolymerization associated with freezing injury in *Allium cepa* root tip cells. Cryo-Letters. 1984;5:373–82.

[39] Olszewska MJ, Kuran H, Marciniak K. Relationship between resumption of rRNA transport into cytoplasm after cold treatment and progression through the cell cycle in root meristem cells. Env Exp Bot. 1988;28(4):375–80.

